# Models for Predicting Drug Absorption From Oral Lipid-Based Formulations

**DOI:** 10.1007/s40610-015-0023-1

**Published:** 2015-10-07

**Authors:** Linda C. Alskär, Christel A. S. Bergström

**Affiliations:** Department of Pharmacy, Uppsala University, Uppsala Biomedical Center, P.O Box 580, SE-751 23 Uppsala, Sweden

**Keywords:** Lipid-based formulations, Prediction, In vitro models, Molecular dynamics simulations, Multivariate data analysis, Formulation performance

## Abstract

In this review, we describe the in vitro tools currently used to identify when a lipid-based formulation has the potential to deliver a poorly water-soluble drug via the oral route. We describe the extent to which these tools reflect the in vivo performance of the formulation and, more importantly, we present strategies that we foresee will improve the in vitro-in vivo correlations. We also present emerging computational methods that are likely to allow large parts of the formulation development to be carried out in the computer rather than in the test tube. We suggest that these computational tools will also improve the mechanistic understanding of in vivo formulation performance in the complex and dynamic environment of the gut.

## Introduction

Many therapeutic targets currently being explored have highly lipophilic endogenous ligands. This biological fact often results in potent drug candidates identified during drug discovery that are highly lipophilic with poor solubility in water [1, 2]. Indeed, most newly discovered compounds belong to this group; there are indications that the solubility of up to 90 % of all new drug molecules is too low to allow the complete dose to be dissolved in the intestinal fluid. The first major hurdle to overcome when such compounds are orally administered is to achieve a high enough concentration to support absorption from the gastrointestinal (GI) tract so that the drugs can reach their targets through the systemic circulation. One method of successfully delivering highly lipophilic compounds is to use lipid-based formulations (LBFs); this method enables lipophilic, poorly water-soluble compounds to be administered orally [3••]. In contrast to conventional oral formulations (e.g. tablets), LBFs typically deliver the drug in a solubilized state, thus bypassing the need for in vivo dissolution. Further, LBFs often present the active pharmaceutical ingredient (API) in a supersaturated state in the intestinal fluid, and the driving force for absorption is thus increased through the increase in API concentration in the GI tract [4, 5].

## Formulation Development

LBFs are complex systems that contain a mixture of lipids, surfactants and/or cosolvents. The loading capacity of the formulation (i.e. the maximum amount of API that can dissolve in the LBF) is dependent on all these components. There are as yet only a few marketed products that have been formulated as orally delivered LBFs; some examples are Sandimmune© and Sandimmune Neoral© (cyclosporin A), Norvir® (ritonavir) and Fortovase® (saquinavir) [6]. In total, about 2–4 % of the marketed products take use of this formulation strategy [7••]. The system developed by Pouton for classifying LBFs into four groups on the basis of their lipophilicity (Table 1) [8••] is used to improve the understanding of the performance of formulated drugs, in particular with regard to the intestinal stability of the supersaturated state in response to enzymatic digestion of, for example, LBF glycerides.[9–11]

**Table 1:** The four classes (I–IV) Included in the Lipid Formulation Classification System [8••]. The following abbreviations are used: tri-, di- and mono-glycerides (TG, DG and MG, respectively); hydrophilicity-lipophilicity balance (HLB); weight per weight percent (%*w*/*w*)

Excipient	Type I %w/w	Type II %w/w	Type IIIa %w/w	Type IIIb %w/w	Type IV %w/w
Oils: TG or mix of MG/DG	100	40–80	40–80	<20	–
Water-insoluble surfactant (HLB < 12)	–	20–60	–	–	0–20
Water-soluble surfactant (HLB > 12)	–	–	20–40	20–50	30–80
Hydrophilic cosolvent	–	–	0–40	20–50	0–50
	Most lipophilic			Most hydrophilic	

**Fig. 1:**
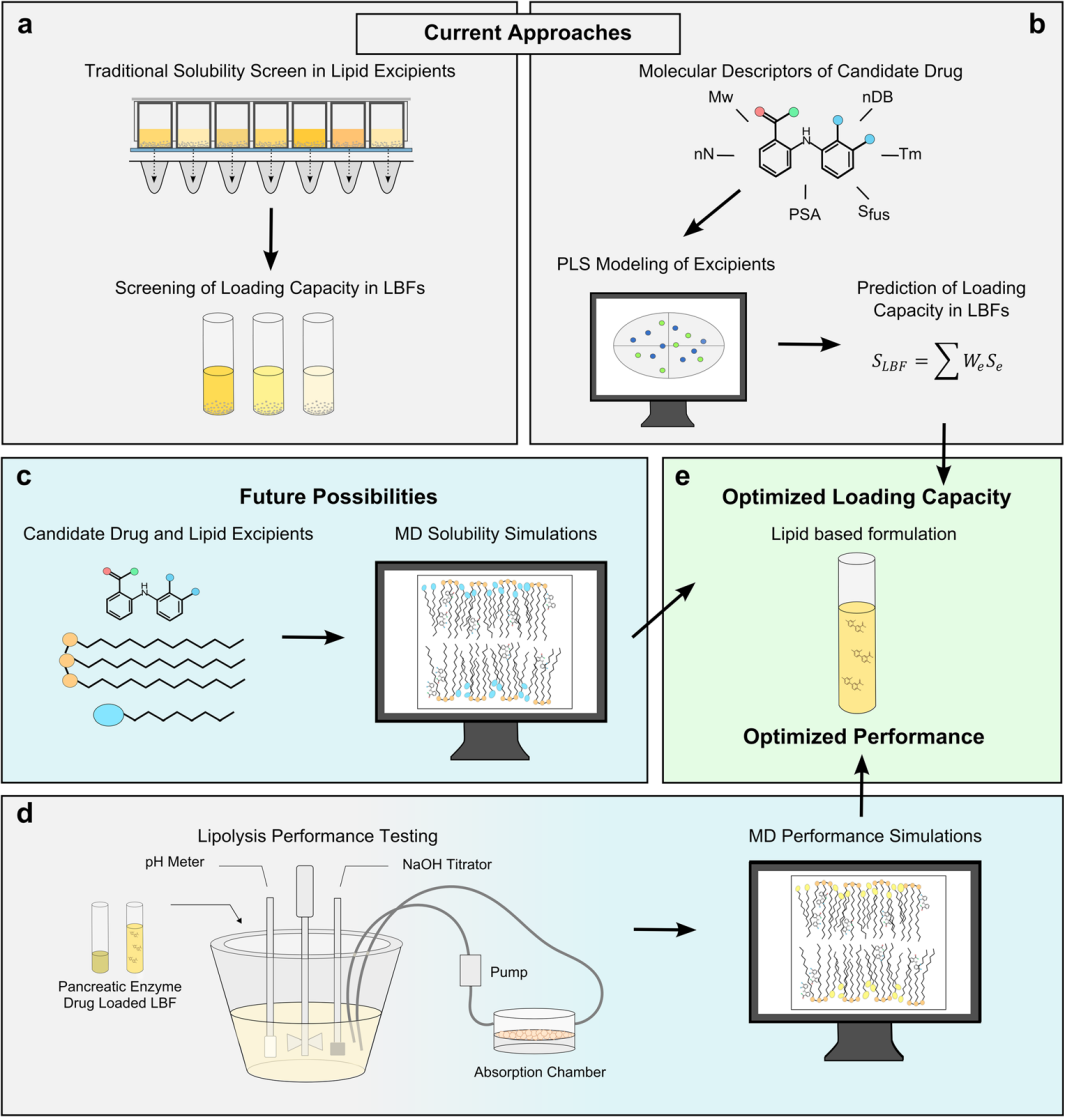
Current and future assessment of the loading capacity (maximum amount of drug that can be dissolved) and in vivo performance of LBFs. **(** *a*) The solubility of the drug in key excipients and the loading capacity of the potential LBFs are currently assessed experimentally, typically in a 96-well titer plate, for a large number of excipients and formulations. This exercise requires a large amount of the compound to be synthesized and may result in sub-optimal formulations to be selected, as only a standard selection of formulations is studied. (*b*) Recently, computational multivariate data analysis models have been developed to allow the drug solubility in excipients and the loading capacity of the formulations to be predicted from calculated molecular descriptors and information obtained from the solid state. (*c*) The use of MD simulations allows molecular interactions between drug and excipients/LBFs to be identified and the free energy of solvation in the formulation to be calculated. These computational simulations are likely to result in more accurate prediction of the solubility and loading capacity and will also increase the mechanistic understanding of the solvation process of the API in complex formulations. (*d*) Novel in vitro and in silico tools to predict the dynamic gut. The lipolysis as performed today is shown on the *left hand side* with the reaction vessel to which the LBF is added. A future goal is to connect this in vitro model with an absorption chamber mimicking the intestinal wall which will allow absorption of API and LBF components to occur simultaneously with the digestion. Furthermore, protocols for MD simulations that assess the impact of dispersion and digestion of the LBF on the solvation capacity of the intestinal fluid need to be developed. These simulations should capture the restructuring of the solubilizing nanoaggregates present in the intestinal fluid to better predict in vivo performance of LBF dosed drugs. (*e*) The end goal is the accurate optimization of loading capacity and in vivo performance using in silico tools.

However, the selection of an LBF is currently largely made by experimentally screening the loading capacities of a large number of differently formulated, commonly used LBFs (Fig. 1a) [12] rather than allowing the physicochemical properties of the API to inform on which type of LBFs that is likely to be successful. This experimental screening exercise requires that a significant amount of the API has been synthesized, which means that evaluation of LBFs as a suitable means for delivering poorly water-soluble APIs cannot be carried out until late in the development stage. Also, it means that non-optimized formulations might be selected for in vivo studies, typically resulting in animal experiments that subsequently fail to reveal any useful pharmacokinetic properties.

## Formulation Performance and Drug Absorption

In vitro, the performance of an LBF is investigated through lipolysis studies [3••, 13]. A standardized in vitro lipolysis experiments is commonly used to mimic the in vivo situation and grade the performance of the LBF (Fig. 1d) [14, 15]. There are several versions of the experimental setup but in general it involves a temperature-controlled (37 °C) vessel that contains a digestion buffer (pH 6.5), bile salts and phospholipids. The drug-loaded formulation is added to the vessel and, after a period of dispersion (to mimic the gastric emptying of the formulation into the duodenum prior to bile secretion), digestion is initiated by the addition of pancreatin. Lipid digestion results in liberation of fatty acids (FAs), which cause the pH to drop. A pH-stat meter is used to monitor the pH because the digestion process is pH-sensitive and it is crucial to compensate for FA liberation. Typically, compensation is achieved by addition of equimolar sodium hydroxide. The pH monitoring and addition of equimolar sodium hydroxide also provides an estimation of the degree and rate of digestion. Samples are taken throughout the digestion process and these are centrifuged to separate the water, oil and pellet phase. The samples are subsequently analysed to determine the drug concentration and thus obtain an estimate of the extent to which the drug has remained in its solubilized form [16]. If the formulation is digestible, enzyme processing can result in loss of the solubilizing capacity of the LBF with subsequent precipitation of the drug and loss of the solubility advantage provided by the original formulation. The extent to which this precipitation is detrimental to absorption of the API depends on the form that precipitates. If the precipitate is the amorphous form, it can be easily redissolved and may not hinder absorption as much as if the thermodynamically stable crystalline polymorph precipitates. Recently, in situ Raman spectroscopy has been used in digestion studies to reveal the extent of precipitation and the form of the precipitated compound [17•, 18] and also to study drug excipient interactions in less complex dispersions [19].

Still, little is known about the in vivo dynamics of the intestinal lipoidal nanostructures formed in response to oral intake of lipid-based medications. These solubilizing nanoaggregates are restructured as a response to dilution upon water intake, digestion and/or absorption of LBF components. Colloidal structures in the duodenum, where the gall bladder secretes bile, are likely to differ significantly from those in the distal part of the jejunum or in the ileum. This colloidal rearrangement often occurs rapidly and can produce a wide variety of lipoidal nanostructures. Recently, using in vitro digestion of milk as an example of food-triggered restructuring of intestinal nanoaggregates, synchrotron small-angle X-ray scattering (sSAXS) identified that the milk fat proceeded from oil droplets through microemulsion of micellar cubic and hexagonal phases to a bicontinuous cubic phase [20]. In another recent study, microscopy was used to visualize the structures present in post-prandial human intestinal fluid 1 h after a meal [21]. As expected, micellar structures were abundant in the fluid as a result of the high levels of phospholipids and bile salts present. In addition to these, a number of uni- to multilamellar structures were found. Hence, the dynamic in vivo situation results in a complex absorption process for LBFs.

It is well known that the in vitro-in vivo correlation (IVIVC) between drug performance in the lipolysis experiment and that after administration to animals is modest. Generally, level C correlations are obtained resulting in a correct rank order of formulations rather than in quantitative predictions [22–25]. One of the main reasons for this is that the in vitro digestion method currently in use does not allow for the absorption of either the API or of the LBF components. Absorption of released free FAs derived during the digestion has been simulated in vitro by addition of calcium to the medium [13]. The calcium forms complexes with the free FAs and precipitates. However, free drug is not transported away from the solution as would be the case for highly lipophilic, freely permeating compounds in vivo. This can cause an irrelevant high supersaturation level in the lipolysis experiment, with resultant driving of precipitation in vitro that would not occur in vivo [22, 26]. Several attempts have been made to mimic the interplay between digestion and absorption; however, the harsh digestion medium which includes surfactants is not well-tolerated by model cells [27, 28]. The few studies in which a digestion medium has been applied to cell monolayers indicate that the toxicity profile of the applied medium will be highly dependent on the formulation investigated.

## Computational Tools for Addressing Drug Loading and Formulation Performance

One of the most important conditions for a successful LBF is that the drug can be adequately dissolved in the lipid system used [29••, 30, 31]. Traditionally, phase diagrams have been used to compose miscible formulations and the loading capacity of the LBF has subsequently been determined [32]. The log-linear model proposed by Yalkowsky and coworkers is an early model to estimate drug loading in cosolvent-based systems. This log-linear model describes the exponential increase in the solubility of non-polar drugs (on a log scale) with the linear increase in cosolvent concentration [33, 34]. Recent studies of lipid systems have used this theory to estimate drug solubility in complex lipid mixtures [35–37]. One of the weaknesses of this methodology is the requirement for experimental measurements, which limits their applicability for rapid estimation of solubility. Efforts to facilitate computational prediction of drug solubility are therefore warranted and can be expected to increase the throughput and lower the costs of LBF development [29••]. As described by Anderson et al. [38], the use of the ideal solubility theory and regular solution theory fail to provide accurate prediction of drug solubility in polar organic lipid solutes and solvents. This is mainly an effect of the absence of molecular interactions in the calculations. Since different multivariate data analysis methods, such as partial least squares projection to latent structures (PLS), artificial neuron network (ANN) and support vector machine/regression (SVM/SVR), have previously been successful in predicting drug solubility in water-based systems (see e.g. [39]), we have recently used multivariate data analysis to predict drug solubility in single excipients [40•]. Our in-house dataset now consists of ~40 compounds determined in nine key LBF excipients. This dataset indicates that computationally predicted solubility values obtained from PLS models based on rapidly calculated molecular descriptors and solid state data can successfully predict loading capacity of complex LBFs when an approach similar to the log-linear model is taken (Fig. 1b) (Alskär et al., unpublished data). Hence, the LBFs that provide sufficient loading of the API can be selected using a computer if solid state characteristics such as the melting point and entropy of fusion are available. It is worth mentioning that these experimental data can be determined rapidly (~10 min) using differential scanning calorimetry and a small amount of solid (~0.5 mg).

Currently, there are no developed computational models or simulation platforms that can be used to reveal the molecular interactions between the API and LBFs (Fig. 1c) or the API and the colloidal systems formed upon dispersion and digestion of the LBFs in intestinal fluids (Fig. 1d). Such a platform would provide insight into food- and formulation-driven solubilization and/or the risk of precipitation in vivo, and could potentially also provide information on population performance and interindividual variability. A suitable means of achieving this could be to calculate the free energy of solvation from first principles, applying molecular dynamics (MD) simulations [41•]. MD simulations have been widely applied in the drug discovery process to reveal potential interactions with biological targets and membranes [42–45]. Significant effort has gone into using MD simulations to improve our understanding of biological membranes, in particular the structure and hydration of different phospholipids [42, 43, 46, 47], with the overall aim of better understanding the transport processes in living cells. MD simulations have also been extensively used to simulate simple surfactant systems.^1^ However, they are still relatively unexplored in the field of drug development, and it is suggested that they would be a suitable tool for predicting formulation performance. A limited number of publications offer data on the spontaneous aggregation of bile acids, pharmaceutically relevant lipids and/or excipients [48–53]. For instance, simulations of the liquid phase behavior of pharmaceutically relevant surfactants have been studied to better understand colloidal interactions [54]. This study used the united-atoms approach and 33 different MD simulations to describe the complete ternary phase diagram of the sodium oleate, sodium laurate and water system. The simulations correctly identified the three phases (micellar, hexagonal, lamellar) found experimentally. Hence, it is clear that the computational power to simulate large, complex structures is available and that MD simulations of systems including naturally available lipids and the excipients of LBFs are feasible.

## Conclusions and Future Outlook

Currently, the use of LBFs as a means of delivering highly lipophilic, poorly water-soluble compounds by the oral route is largely assessed by in vitro tools. The decision on whether to pursue an LBF for a particular API is highly dependent on the experimental screening results for loading capacity and performance during digestion, where the latter studies are investigated under rather simplified conditions in a reaction vessel. While these tools may successfully identify a formulation with the capacity to deliver the required amount of the API, there is a high risk that the selected formulation is not optimal for the drug in question. In particular, the current methods make it difficult to predict the tendency for supersaturation to occur in vivo. In the GI tract, the interplay between the dosage form, the API and the physiology (intestinal fluid composition, enzymatic capacity, permeation through the intestinal wall, gastric emptying and transit times) is determining the in vivo supersaturation and precipitation risk, and this dynamic situation is difficult to capture by the methods currently in use. In addition, these measurements cannot be performed until a substantial amount of the API is available. To make the selection of an LBF more driven by knowledge and to retrieve valuable information on the potential in vivo performance early in the development process, new or refined tools are required for assessing both the loading capacity and the in vivo performance. We have identified three current limitations for which new or refined technological development is essential:

1. *The lack of knowledge about interindividual variability in the composition, size and form of the solubilizing lipid nanoaggregates in the GI tract.* Human intestinal fluid has to be better characterized at an individual level to allow population assessment of LBF performance in the fasted and fed states. These data can then be used as input in computational simulations to develop a virtual gut. New technologies that require only small sample volumes for characterization of colloidal structures are emerging (e.g. in situ/in-line Raman spectroscopy, sSAXS, SANS, UPLC-MS/MS) and these will enable more detailed characterization of colloidal structures present in vivo.
2. *The lack of an absorption compartment in the lipolysis (LBF digestion) assay.* There have been only limited attempts to date to include an absorption compartment in the lipolysis assay; the dispersion, digestion and permeation are studied separately rather than in concert. It is likely that LBF performance will be better predicted by an in vitro tool that enables absorption of both the API and the digested components, as such an in vitro system would better reflect the restructuring and permeation processes present in the dynamic environment that the LBF is exposed to in the intestine.
3. *Resistance to use highly complex computational simulation tools such as MD simulations in formulation assessment.* Clearly, the computational power is available to allow complex systems, such as lipoidal aggregates present in the intestinal fluid, to be simulated. In the future, these tools should be used on a wider basis to explore the impact of different excipients on the intestinal colloidal structures. They would also be suitable for studying the rearrangement of the solubilizing aggregates upon digestion. We suggest that different resolution scales could be used to explore the restructuring as they occur: coarse-grained resolution to identify phase changes in response to dilution or digestion and all-atom scale to explore drug solubilization. Tools that facilitate such a multi-scale molecular modelling approach are currently available (e.g. INSANE, CGTools and Backwards Tools) and these are likely to enable computerized representation of the restructuring of the nanoaggregates. However, it should be noted that more knowledge about the composition of and structures formed in human intestinal fluid (see point 1) would be necessary to succeed in the virtual assessment of colloidal restructuring.

We believe that these suggestions will improve the reliability of the IVIVC for LBFs. More importantly, they have the potential to transform this experimentally demanding research field to predictive science, where computational models inform the formulators of successful delivery strategies. This approach will significantly improve the mechanistic understanding of these complex dosage forms and their interplay with the gut at the molecular level.

## Footnotes

1 A search string of “Molecular dynamic* AND simulation* AND surfactant*” resulted in 604 hits at PubMed on July 22, 2015.
